# Optimal exercise modalities and dose for enhancing intelligence in children and adolescents: a Bayesian network meta-analysis

**DOI:** 10.3389/fphys.2025.1685099

**Published:** 2026-01-15

**Authors:** Yan Wang, Junyu Wang, Lin Zhang, Chengji Wang, Guotuan Wang, Changdong Li, Yuan Yuan, Bopeng Qiu, Yong Yang

**Affiliations:** 1 Laboratory of Kinesiology and Rehabilitation, School of Physical Education and Sport, Chaohu University, Hefei, China; 2 School of Exercise and Health, Shanghai University of Sport, Shanghai, China; 3 Department of Rehabilitation, West China Hospital Sichuan University Jintang Hospital, Jintang First People's Hospital, Chengdu, China; 4 Laboratory of Kinesiology and Rehabilitation, School of Physical Education and Sport, Henan University, Kaifeng, China; 5 Faculty of Physical Education, National Research Tomsk State University, Tomsk Oblast, Russia; 6 Department of Sport Industry Studies, Yonsei University, Seoul, Republic of Korea; 7 Division of Sports Science and Physical Education, Tsinghua University, Beijing, China

**Keywords:** adolescents, children, dose-response relationship, exercise, intelligence

## Abstract

**Background:**

To compare the effects of various exercise modalities on intelligence and determine the optimal exercise dose for children and adolescents.

**Methods:**

A systematic review and Bayesian network meta-analysis following PRISMA guidelines was conducted. Four databases were searched up to 1 April 2025. Eligible RCTs involved participants aged 5–18 years and assessed exercise interventions with intelligence outcomes (general, fluid, or crystallized). Standardized mean differences (SMDs) with corresponding 95% confidence interval or credible intervals were calculated. Dose-response relationships were analyzed using model-based network meta-analysis.

**Results:**

Fifteen RCTs with 3,400 participants were included. Exercise was linked to small-to-moderate improvements in general (SMD = 0.59), fluid (SMD = 0.43), and crystallized intelligence (SMD = 0.64). Dual-task balance training (DTBT) produced the most consistent and significant benefits across all domains. Yoga and multi-component exercise also showed positive effects. Optimal outcomes were achieved with sessions lasting ≥117.7 min, three times weekly, totaling 220 min per week for at least 11.12 weeks. An inverted U-shaped dose-response curve indicated diminishing returns beyond the optimal frequency and duration.

**Conclusion:**

DTBT is the most effective exercise modality for improving intelligence in children and adolescents. The findings provide evidence-based guidance for designing school and clinical exercise programs to support cognitive development during growth.

## 1 Introduction

Intelligence, defined as the capacity for logical reasoning, abstract thinking, problem-solving, and adaptive learning, constitutes a foundational determinant of cognitive, academic, and socioemotional functioning across the lifespan (Hearne et al., 2016). In children and adolescents, intelligence plays a pivotal role in shaping academic trajectories, social competence, and the capacity to navigate future challenges (Duckwort et al., 2019). Although these formative years represent a critical window for interventions to optimize intelligence, relatively few studies have focused specifically on intelligence development in this population. Existing research has often examined broader cognitive outcomes or scholastic performance, leaving notable gaps in our understanding of how to enhance intelligence itself (Kievit et al., 2017; Watson et al., 2022; Wang et al., 2025). Given the potential for significant long-term benefits, further inquiry into targeted strategies for fostering intelligence in youth is urgently needed.

A variety of factors influence intelligence in young populations, including genetic predispositions, educational environments, social contexts, and health behaviors (Plomin and von Stumm, 2018). Among these factors, exercise stands out as a behavior-based intervention that can be implemented systematically within school or community settings. Cultivating active habits during childhood and adolescence may yield lifelong advantages for both cognitive and intellectual development (Chen et al., 2020). Existing research suggests that various forms of exercise may foster improvements in brain health, such as enhanced plasticity and vascularization, which could translate into gains in intelligence (Erickson et al., 2019; Hillman et al., 2008). Although additional rigorously designed trials are needed to establish causal links, the potential to modify exercise levels and patterns makes it an attractive target for interventions aimed at improving intelligence and learning capacity in children and adolescents.

In light of these findings, an increasing number of studies have shifted focus from broadly defined exercise to more structured and organized exercise in an effort to clarify their specific effects on intelligence in children and adolescents. While several meta-analyses have been conducted to synthesize the impact of exercise on cognitive outcomes, the majority have concentrated on general cognitive functions rather than intelligence *per se* (de Greeff et al., 2018; Wang et al., 2023; Sun et al., 2021; Wang et al., 2024). To date, one meta-analysis has reported that regular exercise may exert a positive effect on intelligence in children and adolescents (Morales et al., 2024). However, this analysis did not distinguish between different types of exercise, thereby leaving unresolved the question of which specific modalities are most effective. Furthermore, it did not investigate dose-response relationships in adequate detail, limiting the capacity to derive precise, evidence-based recommendations for optimizing intervention strategies. These methodological limitations underscore the need for more comprehensive evaluations to inform the development of targeted, evidence-based exercise protocols aimed at enhancing intelligence during critical developmental periods.

To determine the optimal exercise modalities for intelligence improvement (Shephard, 1997). The present study aims to address these gaps by employing a Bayesian network meta-analysis to compare the effects of various exercise modalities on distinct domains of intelligence in children and adolescents. In addition, dose-response models are applied to identify the optimal exercise frequency, session duration, and total weekly volume required to maximize cognitive benefits. By integrating these two analytical approaches, the study seeks to determine the most effective exercise strategies for promoting intellectual development in youth and to elucidate the dose-response relationships that underlie these effects. The findings are intended to provide educators, healthcare professionals, and policymakers with precise, evidence-based recommendations for designing and implementing optimized exercise interventions to enhance intelligence during critical developmental stages.

## 2 Methods

### 2.1 Protocol and registration

This pre-registered systematic review and network meta-analysis, adhered to the reporting requirements outlined in the PRISMA checklist (Page et al., 2021) and was registered in PROSPERO (registration number: CRD420251023092).

### 2.2 Literature search

We conducted an extensive search across several databases—PubMed, Embase, Cochrane Central Register of Controlled Trials (CENTRAL), and Web of Science—covering their entire history up until 1 April 2025, without any language restrictions. The full search strategies, including terms, dates, and methods, are outlined in Supplementary File 1. Additionally, we reviewed the reference lists of relevant articles and reviews to identify further studies. Title, abstract, and full-text screening were carried out independently and in duplicate by researchers. Any discrepancies were resolved through discussion or, if needed, by consulting a third author for final resolution.

### 2.3 Eligibility criteria

In accordance with the PICOS approach (Hutton et al., 2015), the inclusion criteria were as follows: (a) participants: children and adolescents aged 5–18 years old; (b) intervention: exercise in different forms (Supplementary File 2); (c) comparator: non-physically active control groups (e.g., no intervention, maintained their regular classroom schedules, or waitlist) (CON) were included. For head-to-head studies, the comparator could be any type of exercise different from that of the experimental group; (d) outcomes: analyzed various forms of intelligence measures and their specific domains, including general intelligence, crystallized intelligence, and fluid intelligence; (e) study design: included published RCTs (Liberati et al., 2009). Specifically, we included studies in which the effect of exercise could be isolated. For instance, for studies in which the exercise group combined exercise with another training intervention, the study was included only if the control group received the same intervention without the exercise component. Moreover, we excluded studies on the acute effects of a single exercise session on children and adolescents, and studies that did not clearly describe the specific exercise variables (e.g., period, frequency, volume). Additionally, studies that did not analyze all subtests covered by the intelligence test used were excluded.

### 2.4 Data extraction

After retrieving all relevant articles from the specified databases, they were organized in an EndNote X9 reference manager. Two authors independently conducted data extraction from studies that met the inclusion criteria, resolving any discrepancies through consensus among all authors. The extracted data included pertinent publication details (such as author, title, year, and journal), sample size, demographics (e.g., age and sex), and descriptions of the interventions. If the original study reported standard errors for the experimental and control groups, the standard deviation (SD) was calculated using the formula: $\text{standard deviation}\left(\text{SD}\right)=\text{standard error}\left(\text{SE}\right)*\sqrt{n}$ . In cases where both standard deviation and standard error were unavailable, SD estimation was performed using other available parameters such as confidence intervals, *t*-values, quartiles, ranges, or *p*-values (Higgins and Green 2008). If essential data could not be derived through these methods, attempts were made to contact the study authors up to four times within a six-week period to request the missing information.

### 2.5 Risk of bias and quality of evidence

Two authors evaluated the risk of bias in the studies using the revised Cochrane risk of bias tool (RoB 2 tool) at the study level (Sterne et al., 2019). Any disagreements in data extraction or assessment were resolved by consulting a third reviewer. We also examined the confidence of evidence using the CINeMA (Confidence of Network Meta-Analysis) web application, which allows the confidence of the results to be graded as high, moderate, low, and very low (Nikolakopoulou et al., 2020).

### 2.6 Data synthesis and analysis

#### 2.6.1 For pairwise meta-analysis

We combined studies with a CON to assess whether exercise is effective in improving intelligence in children and adolescents. To this end, we applied Hedges’ *g* to calculate the between-subject standardized mean difference (SMD_bs_) ( $SMD_{bs}=\frac{Exercise\;\left(M_{change}\right)-CON\;\left(M_{change}\right)}{{SD}_{pooled}}$ ), where the *SD*_pooled_ was calculated as: ${SD}_{pooled}=\sqrt{\frac{{\left(n_{1}-1\right)\;SD}_{1}^{2}+{\left(n_{2}-1\right)\;SD}_{2}^{2}}{\left(n_{1}+n_{2}-2\right)}}$ . The SMD_bs_ was then adjusted for the sample size by using the term $\left(1-\frac{3}{4N-9}\right)$ (Hedges et al., 1985), where *N* is the total sample size of the exercise group and control groups, where *n*_1_ is the sample size of the exercise group and *n*_2_ is the sample size of the control group. The amount of pre and post change of the exercise group and control group were calculated by the following formula: $M_{change}=M_{Post}-M_{Pre}$ ; $SD_{change}=\sqrt{SD_{Pre}^{2}+SD_{Post}^{2}-\left(\;2*r*SD_{Pre}*SD_{Post}\right)}$ , where R is a constant (*r* = 0.5) (McKenzie and Brennan, 2019). Cohen’s criteria were used to interpret the magnitude of SMD_bs_: <0.5, small; 0.5 to 0.8, moderate; and >0.8, large (Fisch et al., 1988).

#### 2.6.2 For network meta-analysis

A network plot was generated to visually represent the network of comparisons across trials, ensuring the viability of the network meta-analyses. In the context of comparing the effects of various exercise types, we conducted Bayesian network meta-analyses using the ‘gemtc’ and ‘rjags’ packages within the R statistical environment (V.4.2.2, www.r-project.org). This approach involves calculating the posterior distribution of parameters based on the available data to update prior information, as Bayesian methods are more prevalent in such analyses compared to frequentist approaches. Markov chains were used to generate samples. Model convergence was assessed using the Brooks-Gelman-Rubin plots method. The effect sizes were calculated as standardized mean differences (SMDs) of the change score because the studies use different rating scales or units of the outcome. To evaluate the reliability of our estimates, we utilized 95% credible intervals (CrI). For data synthesis, a random-effects model was employed to combine the data, while the surface under the cumulative ranking (SUCRA) probabilities was utilized to rank the various exercise types. Statistical heterogeneity between studies was examined using the tau-square (τ^2^) test and I^2^ statistics. Statistical consistency was evaluated using the design-by-treatment test (Higgins et al., 2012) and by differentiating indirect from direct evidence (SIDE test) (Dias et al., 2010) via the R ‘netmeta’ package (Supplementary File 5). Publication bias was assessed using contour-enhanced funnel plots and Egger’s test, with *p* < 0.05 indicating significant bias (Supplementary File 6).

#### 2.6.3 For dose–response analysis

The ‘MBNMAdose’ package based on R statistical environment (V.4.2.2, www.r-project.org) was used to perform Bayesian Model-Based Network Meta-Analysis (MBNMA) (Mawdsley et al., 2016) to summarize the dose-response association between exercise dose (period, frequency, weekly exercise time and single course duration) and intelligence. In order to better find the relationship between exercise dose and intelligence, a series of nonlinear and linear models were used. Next, we derived and compared different fit indices and corresponding deviance plots across all estimated models (Evans, 2019). The Emax prediction model was used for single course time and period, and the restricted cubic spline model was used for weekly exercise time and frequency (Supplementary File 8).

## 3 Results

**FIGURE 1:**
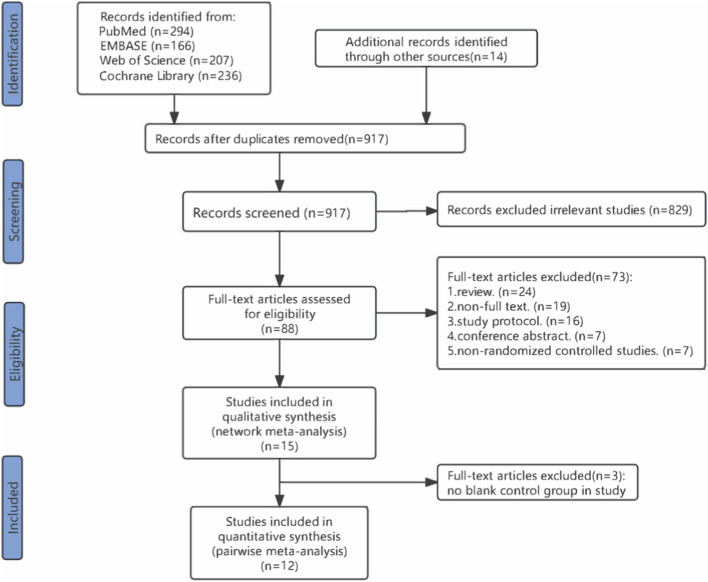
PRISMA flow diagram of the search process for studies.

Overall, 917 records were identified through the initial electronic searches. 829 articles were excluded due to duplication or through evaluation of titles and abstracts, and 88 full-text articles were screened for eligibility. In total, 15 studies involving 3,400 participants were included in the systematic review (Figure 1).

### 3.1 Characteristics of included studies

The characteristics of included studies were shown in (Table 1). Three studies compared the effects of different types of exercise on intelligence in children and adolescents (Chaya et al., 2012; Jia et al., 2021; Parajuli et al., 2022), and the remaining 12 studies included a non-exercise control group (Ardoy et al., 2014; Atak et al., 2023; Chaddock-Heyman et al., 2013; Corder, 1966; Fedewa et al., 2015; Fisher, 1971; Hirata, 2023; Ortega et al., 2022; Reed et al., 2010; Rymarczyk et al., 2024; Sánchez-López et al., 2019; Uma et al., 1989). A total of 197 participants across 3 studies underwent aerobic exercise (AE) (Jia et al., 2021; Fedewa et al., 2015; Rymarczyk et al., 2024), a study of 30 participants involved balance training (BT) and dual task balance training (DTBT) (Atak et al., 2023), 10 studies with 1,706 participants underwent multi-component exercise (Mul_C), a study with 43 participants underwent perceptual motor training (PMT), 3 studies with 184 participants underwent yoga. The sample size of the included studies ranged from 16 to 1,810. The mean age ranged from 5.1 to 14.5. The year of publication ranged from 1966 to 2024. The exercise lasted for 4–40 weeks, with a frequency of 2–5 times/week, 20–76.8 min per session. Very few studies reported detailed information on the intensity of exercise.

**TABLE 1:** Characteristics of studies and subjects included in the review.

Study	Current status	Country	Mean age	Sample size (girl)	Intervention detail
Ardoy et al. (2014)	Normal	Spain	Mul_C1: 12.9 (0.51)	26 (NA)	Regular physical education courses (strength, aerobic, coordination, and flexibility exercises), 55 min, four per week, moderate intensity, 16 weeks
			Mul_C2: 12.7 (0.48)	23 (NA)	Regular physical education courses (strength, aerobic, coordination, and flexibility exercises), 55 min, four per week, high intensity, 16 weeks
			CON: 13.8 (0.42)	18 (NA)	Remained in the classroom and received the usual classroom instruction
Chaddock-Heyman et al. (2013)	Normal	United States	Mul_C: 8.9 (0.7)	14 (7)	Cardiorespiratory endurance, muscular strength, motor skills and game play. 76.8 min, five per week, moderate to high intensity, 36 weeks
			CON: 8.9 (0.4)	9 (6)	Wait list
Corder (1966)	Educable mentally intellectual disability boys	United States	Mul_C: 12–17	8 (0)	Received a progressive and systematic program of physical education., 60 min, 5 per week, 4 weeks
			CON: 12–17	8 (0)	Remained in the classroom and received the usual classroom instruction
Fisher (1971)	Educable mentally intellectual disability boys	United States	PMT: 9.5 (1.22)	16 (NA)	Perceptual motor training (Improved coordination and motor skills, enhanced cognitive function), 2 per week, 13 weeks
			CON: 10.0 (0.81)	16 (NA)	Maintained their regular classroom schedules
Ortega et al. (2022)	Overweight and obesity	Spain	Mul_C: 9.99 (1.13)	57 (20)	Aerobic, strength training and coordinative exercises, 60 min, 3–5 per week, moderate to high intensity, 20 weeks
			CON: 10.10 (1.13)	52 (25)	Continued their usual routines
Hirata (2023)	Normal	Brazil	Mul_C: 5.1 (0.6)	1,256 (NA)	Capoeira, 40 min, 3 per week, 20 weeks
			CON: 5.1 (0.6)	554 (NA)	Wait list
Atak et al. (2023)	Mild and borderline intellectual disabilities	Turkey	DTBT: 8.87 (1.45)	15 (NA)	Dual task balance exercise, 30 min, 2 per week, 12 weeks
			BT: 8.20 (1.52)	15 (NA)	Standard balance exercise, 30 min, 2 per week, 12 weeks
			CON: 8.93 (1.66)	15 (NA)	Cognitive training
Parajuli et al. (2022)	Normal	India	Yoga: 12.71 (0.67)	43 (43)	Yoga (breathing exercises, asana (yogic postures), pranayama (yogic breathing exercises), and dhyana (meditation)), 60 min, 4 per week, 8 weeks
			Mul_C: 12.60 (0.76)	43 (43)	Physical exercise (jogging, stretching, and joint loosening exercises as well as outdoor games), 60 min, 4 per week, 8 weeks
Reed et al. (2010)	Normal	United States	Mul_C: 9.42 (0.52)	80 (24)	Fundamental skills (i.e., hopping, skipping, jumping, running), 30 min, 3 per week, 13 weeks
			CON: 9.50 (0.53)	75 (33)	Wait list
Sánchez-López et al. (2019)	Normal	Spain	Mul_C: 5.78 (0.42)	82 (48)	Traditional playground games, 60 min, moderate‐vigorous intensity, 3 per week, 32 weeks
			CON: 5.87 (0.35)	158 (87)	Health education
Uma et al. (1989)	Educable mentally intellectual disability boys	India	Yoga: 10.7 (2.7)	45 (16)	Yoga, 60 min, 5 per week, 40 weeks
			CON: 10.8 (2.5)	45 (16)	Wait list
Chaya et al. (2012)	Normal	India	Yoga: 7.8 (0.9)	96 (48)	Yoga, 45 min, 5 per week, 12 weeks
			Mul_C: 7.6 (0.8)	97 (44)	Physical activity (passive stretching and aerobic exercises, such as running and group games), 45 min, 5 per week, 12 weeks
Rymarczyk et al. (2024)	Normal	Poland	AE: 9–11 years	22 (NA)	Aerobic training, 45 min, 5 per week, moderate to high intensity, 10 weeks
			CON: 9–11 years	20 (NA)	Unmoderated free play
Jia et al. (2021)	Normal	China	Mul_C: 6.5 (0.3)	20 (10)	Fundamental skills (i.e., hopping, skipping, jumping, running), 60 min, 3 per week, moderate intensity, 12 weeks
			AE: 6.1 (0.2)	20 (10)	Aerobic training, 60 min, 3 per week, low, 12 weeks
Fedewa et al. (2015)	Normal	UAS	AE: 9–11 years	155 (NA)	Aerobic training, 20 min, 5 per week, 32 weeks
			CON: 9–11 years	297 (NA)	Maintained their regular classroom schedules

**FIGURE 2:**
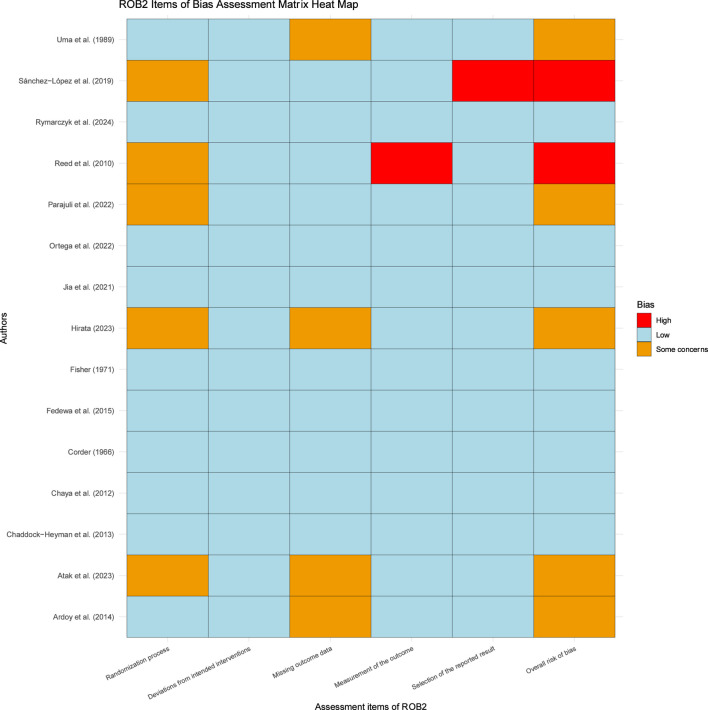
Summary of quality assessment results of included literature.

Of the 15 trials, for overall bias, 8 studies were assessed as low risk of bias, 5 as some concerns and 2 as high. In the randomization process, 10 trials were at low risk of bias, 5 trials were at some concerns risk of bias; for deviations from intended interventions, 15 trials were at low risk of bias; in the missing outcome data, 11 trials were at low risk of bias, 4 trials were at some concerns risk of bias; in the measurement of the outcome, 14 trials were at low risk of bias, 1 trial was at high risk of bias; in the selection of the reported result, 14 trials were at low risk of bias, 1 trial weas at high risk of bias (Figure 2, Supplementary File 3).

### 3.2 Pairwise meta-analysis

A total of 10 effect sizes in 8 studies with 571 participants showed changes in general intelligence. There were moderated effects of exercise in improving general intelligence (SMD = 0.59; 95% CI: 0.42 to 0.76; I^2^ = 0%). A total of 17 effect sizes in 9 studies with 2,432 participants showed changes in fluid intelligence that demonstrated small improvement (SMD = 0.43; 95% CI: 0.35 to 0.52; I^2^ = 34.8%). A total of 9 effect sizes in 5 studies with 238 participants showed changes in crystallized intelligence that demonstrated moderate improvement (SMD = 0.64; 95% CI: 0.01 to 1.27; I^2^ = 33.5%) (Supplementary File 4).

### 3.3 Network meta-analysis

After network meta-analysis of all outcomes, the heterogeneity was significantly reduced. For example, the heterogeneity of general intelligence and fluid intelligence was I^2^ = 0%, and crystallized intelligence was I^2^ = 4.7%. The results of design-by-treatment interaction test and the SIDE test showed that global and splitting inconsistency was not significant (*p* > 0.05) (Supplementary File 5). Additionally, our comparison-adjusted funnel plot had good symmetry for all outcomes, and the results of Egger’s test (*p* > 0.05) showed that no small study effect was found (Supplementary File 6). The confidence of the evidence of 61.9% for general intelligence was very low, 28.6% was low, and 9.5% was moderate. For fluid intelligence, 52.4% was very low, 19.05% was low, 14.3% was moderate, and 9.5% was high. For crystallized intelligence, 57.1% was very low, and 42.9% was low (Supplementary File 9).

Supplementary File 7 showed the direct comparison and sample size distribution between the exercise types for outcomes. 11 of the studies with 804 participants assessed general intelligence. Compared with the CON, 3 of 6 exercise types significantly improved general intelligence, with DTBT (SMD = 0.93; 95% CrI: 0.19–1.68), Mul_C (SMD = 0.60; 95% CrI: 0.42–0.79), and Yoga (SMD = 0.58; 95% CrI: 0.30–0.85) (Supplementary File 7, 7.1). The results of pairwise comparisons showed that DTBT was significantly better than BT. 12 of the studies with 2,751 participants assessed fluid intelligence. Compared with the CON, 3 of 6 exercise types of significantly improved fluid intelligence, with DTBT (SMD = 0.97; 95% CrI: 0.23–1.72), Yoga (SMD = 0.54; 95% CrI: 0.29–0.79), and Mul_C (SMD = 0.50; 95% CrI: 0.41–0.59) (Supplementary File7, 7.2). The results of pairwise comparisons showed that DTBT and Yoga were significantly better than AE. 7 of the studies with 471 participants assessed crystallized intelligence. Compared with the CON, 3 of 6 exercise types significantly improved crystallized intelligence, with DTBT (SMD = 1.05; 95CrI: 0.28–1.82), Mul_C (SMD = 0.70; 95% CrI: 0.44–0.97), and Yoga (SMD = 0.62; 95% CrI: 0.21–1.04) (Supplementary File 7, 7.3). The results of pairwise comparisons showed that DTBT was significantly better than BT.

### 3.4 Dose-response relationships

**FIGURE 3:**
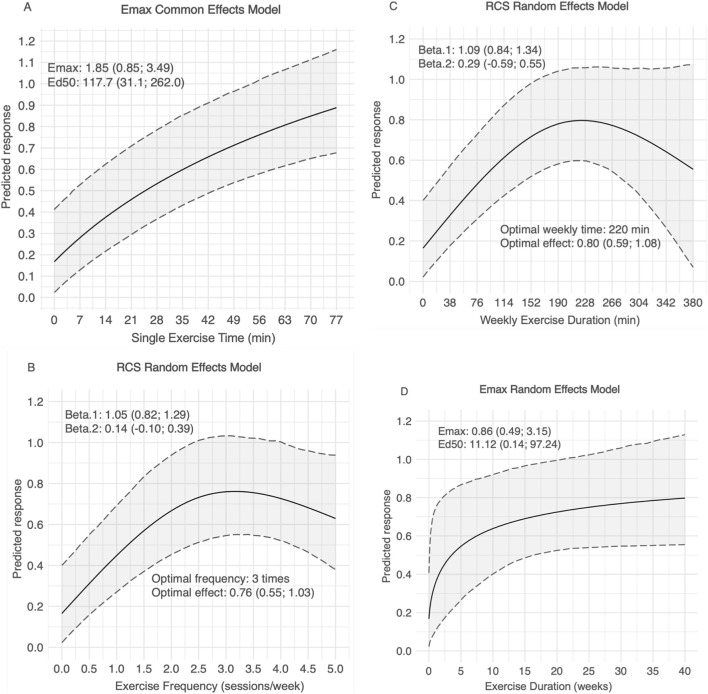
The results of dose-response analysis for general intelligence. **(A)** Single Exercise Time, **(B)** Exercise Frequency, **(C)** Weekly Exercise Duration, **(D)** Exercise Duration.

Figure 3 demonstrated the dose-response relationships between different exercise specific variables and general intelligence. For the single course duration, we used the Emax fixed effect model to fit the data. The results showed that the general intelligence of children and adolescents would increase nonlinearly with the duration of a single course, with Emax = 1.85 (0.85; 3.49), which is the maximum effect of simply increasing the duration of a single exercise course on children and adolescents. In addition, Ed50 (the duration of a single course to achieve 50% of the maximum effect) was estimated to be 117.7 (31.1; 262.0), indicating that a single course duration of approximately 117.7 min may significantly improve the general intelligence of children and adolescents (Figure 3A). We used restricted cubic random effects models to fit the relationship between weekly training frequency and general intelligence in children and adolescents (Figure 3B). The results showed that exercise three times a week was most effective in improving general intelligence in children and adolescents (SMD = 0.76; 95% CrI: 0.55–1.03). At the same time, we also used restricted cubic spline random effects models to fit the relationship between weekly exercise time and general intelligence of children and adolescents (Figure 3C). The results showed that exercise 220 min per week was most effective in improving general intelligence in children and adolescents (SMD = 0.80, 95% CrI: 0.59–1.08). For the exercise period, we used the Emax random effects model to fit the data. The results showed that the general intelligence of children and adolescents would increase nonlinearly with the exercise period, with Emax = 0.86 (0.49; 3.15), and Ed50 was estimated to be 11.12 (0.14; 97.24) (Figure 3D).

## 4 Discussion

This network meta-analysis integrated data from 15 RCTs, encompassing a total of 3,400 children and adolescents, to compare the effects of various exercise modalities on distinct intelligence outcomes. Several noteworthy findings emerged. First, the pooled results revealed a small to moderate overall effect of exercise on intelligence, with moderate improvements in general intelligence, small yet significant gains in fluid intelligence, and moderate enhancements in crystallized intelligence. Second, the network meta-analysis indicated that DTBT consistently produced the greatest benefits across different dimensions of intelligence, whereas yoga and Mul_C also conferred substantial improvements. Third, the dose-response analyses identified a single-session duration of at least 117.7 min, a frequency of three sessions per week, and a total weekly activity time of 220 min as optimal parameters; the findings further suggest that a minimum intervention period of 11.12 weeks is desirable to achieve robust gains in intelligence. Collectively, these results provide a compelling evidence base for designing targeted, evidence-driven exercise interventions to enhance multiple facets of intelligence in children and adolescents, thereby informing future research and practice in both educational and clinical settings.

Intelligence in children and adolescents is frequently conceptualized as a multifaceted construct encompassing general intelligence, fluid intelligence, and crystallized intelligence, each serving distinct developmental purposes (Thapaliya et al., 2025). General intelligence reflects overarching cognitive capacity and learning ability, fluid intelligence pertains to reasoning and the capacity to tackle novel problems, and crystallized intelligence denotes the accumulation of knowledge and skills gained through experience (Scherrer et al., 2024). In the present study, the overall influence of exercise on these domains ranged from small to moderate, with a notably greater impact on general and crystallized intelligence compared with fluid intelligence. This finding resonates partly with earlier meta-analyses that reported positive outcomes of exercise on broad cognitive or academic indicators in youth, although the consistency of effects across different intelligence dimensions has varied among studies (Morales et al., 2024). One possible explanation for the more pronounced benefits observed in general and crystallized intelligence lies in the prolonged engagement required for skill mastery and knowledge acquisition, which may be fostered by structured exercise programs that encourage perseverance, strategic thinking, and goal-directed behavior. Such programs can also facilitate neurobiological adaptations, including enhanced neurotrophin production and synaptic plasticity, thereby creating an enriched milieu for efficient information processing (Borji et al., 2023; St et al., 2018). These mechanisms collectively suggest that establishing well-designed exercise regimens has the potential to strengthen foundational cognitive structures in children and adolescents, ultimately broadening their capacity to acquire and apply knowledge throughout their formative years.

Building upon the insights gained from the pairwise meta-analytic assessments, the network meta-analysis conducted in this study enabled a more nuanced comparison of different exercise modalities and their effects on distinct facets of intelligence in children and adolescents. This comprehensive approach led to another major finding, namely that DTBT emerged as the most efficacious intervention across general, fluid, and crystallized intelligence domains. Although the limited number of previous studies examining DTBT precludes direct comparisons with a broad range of interventions, our results align with emerging evidence suggesting that training protocols combining both cognitive and postural tasks may offer unique advantages over more traditional exercise regimens (Atak and Algun, 2022; Ali et al., 2022). DTBT is characterized by the integration of balance-oriented tasks—such as maintaining a specific stance or engaging in dynamic stability exercises—while simultaneously performing a cognitively demanding secondary task. This dual demand appears to stimulate higher-level cognitive processes, including attentional control, executive functioning, and sensorimotor integration (Lüder et al., 2018). From a mechanistic standpoint, the heightened mental workload in DTBT is hypothesized to strengthen neuroplastic adaptations by engaging cortical regions associated with both motor coordination and cognitive regulation, potentially leading to more robust gains in overall intellectual functioning (Herold et al., 2018). Additionally, the repeated exposure to complex, multi-component tasks may encourage improvements in problem-solving strategies and sustained focus, which could partly explain the broad, cross-domain benefits observed in our meta-analysis (Karbach and Verhaeghen, 2014). By situating DTBT within a wider network of exercise interventions, our findings provide a compelling rationale for incorporating dual-task balance exercises into clinical and educational programs aimed at optimizing intelligence in children and adolescents.

Building upon the identification of the most effective exercise modalities, the present study further explored the dose-response relationships between key exercise parameters and improvements in intelligence, aiming to inform the design of optimal intervention strategies for children and adolescents. The analysis revealed that exercise interventions are most effective when each session lasts at least 117.7 min, occurs three times per week, accumulates to approximately 220 min of weekly activity, and is sustained for a period of no less than 11.12 weeks. A particularly noteworthy finding was the inverted U-shaped relationship observed for both weekly frequency and total weekly exercise time. These patterns suggest that there may be a threshold beyond which increased volume or frequency of exercise ceases to produce additional gains in intelligence and may even be counterproductive. In children and adolescents, whose neurodevelopmental systems are still maturing, excessive training may lead to physical fatigue, psychological stress, and reduced time for academic, social, or unstructured cognitive activities (Brenner et al., 2024). These factors could offset the positive neural and behavioral effects typically associated with moderate exercise, thus limiting the intellectual benefits when activity exceeds optimal levels.

In contrast, a more linear and positive relationship was observed for both single-session duration and total intervention period. Longer sessions may allow for more immersive engagement with complex physical and cognitive tasks, promoting sustained activation of brain regions involved in executive control, attention regulation, and abstract reasoning. Similarly, longer intervention durations may facilitate the accumulation and reinforcement of neural adaptations over time, supporting the consolidation of skills and the long-term development of intellectual capacity (Shi and Feng, 2022). Given that intelligence reflects both the ability to solve novel problems and the acquisition of knowledge, repeated and prolonged exposure to enriched exercise environments may be particularly effective in stimulating both fluid and crystallized aspects of intelligence.

The present study offers several noteworthy strengths. First, the use of a Bayesian network meta-analysis framework enabled a comprehensive evaluation of multiple exercise modalities, which provided nuanced comparisons that extend beyond traditional pairwise methods. Second, the integration of dose-response modeling allowed for the identification of optimal intervention parameters, including session duration, weekly exercise frequency, total weekly exercise time, and exercise duration. This dual-analytic approach furnishes a clearer, evidence-based pathway toward designing exercise programs targeted at enhancing intelligence in children and adolescents. Despite these strengths, some limitations should be acknowledged. First, although the included trials covered a considerable range of participant ages, geographical regions, and intervention protocols, some categories of exercise were still supported by relatively few studies. This limitation may reduce the generalizability of the findings to other populations or settings. Second, the measurement of intelligence varied across studies, with some employing different versions or subtests of standardized instruments, potentially introducing heterogeneity into the pooled effect sizes. Third, data on the long-term sustainability of intelligence improvements were limited. Future investigations would benefit from longitudinal follow-up to determine whether the observed benefits of exercise on intelligence persist over more extended periods. Finally, CINeMA assessments indicated that the overall certainty of evidence for most outcomes was low to very low, underscoring the need for future high-quality studies to further validate these findings.

## 5 Conclusion

This study demonstrated that exercise confers small-to-moderate benefits on intelligence in children and adolescents, with dual-task balance training providing the most pronounced improvements across general, fluid, and crystallized domains. Structured programs of at least 117.7 min per session, conducted three times weekly for a total of 220 min, and sustained for over 11 weeks were associated with optimal outcomes. These findings underscore the importance of well-designed exercise interventions in enhancing both knowledge acquisition and problem-solving skills, highlighting the pivotal role of exercise for intellectual development during formative years. Educators, clinicians, and policymakers should consider integrating targeted, evidence-based activity strategies to maximize intelligence in youth.

## Data Availability

The original contributions presented in the study are included in the article/Supplementary Material, further inquiries can be directed to the corresponding author.
