# Supplementary material for: Optimal exercise modalities and dose for enhancing intelligence in children and adolescents: a Bayesian network meta-analysis
Source: Front Physiol. 2026 Jan 15;16:1685099. doi: 10.3389/fphys.2025.1685099 (PMC12851973; doi:10.3389/fphys.2025.1685099)
Supplement: Supplementary file 1 [file DataSheet1.docx]

**Supplementary**

Table of Contents:

[Supplementary 1: Search Strategy 2](#_Toc194944046)

[Database: PubMed <inception to April 1 2025> 2](#_Toc194944047)

[Database: Embase <1974 to April 1 2025> 5](#_Toc194944048)

[Database: Cochrane 6](#_Toc194944049)

[Database: Web of Science <1965 to April 1 2025> 7](#_Toc194944050)

[Supplementary 2 Definitions and abbreviations of physical activity in the included literature 8](#_Toc194944051)

[Supplementary 3: Risk of Bias 9](#_Toc194944052)

[Table 3 The risk of bias assessment for the individual included studies 9](#_Toc194944053)

[Supplementary 4: Pairwise meta-analysis 11](#_Toc194944054)

[Supplementary 5: Details of SIDE splitting results 12](#_Toc194944055)

[Supplementary 6: Publication bias 15](#_Toc194944056)

[Supplementary 7:The results of network meta-analysis 18](#_Toc194944057)

[Supplementary 8: Non-linera and linear functions and models fit comparison 24](#_Toc194944058)

[Supplementary 9: Grading the evidence of the network meta-analysis using CINeMA 28](#_Toc194944059)

[9.1 CINeMA for the effects of general intelligence 30](#_Toc194944060)

[9.2 CINeMA for the effects of fluid intelligence 31](#_Toc194944061)

[9.3 CINeMA for the effects of crystallized intelligence 32](#_Toc194944062)

# Supplementary 1: Search Strategy

## Database: PubMed <inception to April 1 2025>

***Search Strategy:***

| **Search number** | **Search Details** | **Results** |
| --- | --- | --- |
| 13 | (("Exercise"[MeSH Terms] OR ("Exercises"[Title/Abstract] OR "exercise physical"[Title/Abstract] OR "exercises physical"[Title/Abstract] OR "physical exercise"[Title/Abstract] OR "physical exercises"[Title/Abstract] OR "exercise aerobic"[Title/Abstract] OR "aerobic exercise"[Title/Abstract] OR "aerobic exercises"[Title/Abstract] OR "exercises aerobic"[Title/Abstract] OR "exercise isometric"[Title/Abstract] OR "exercises isometric"[Title/Abstract] OR "isometric exercises"[Title/Abstract] OR "isometric exercise"[Title/Abstract] OR "exercise training"[Title/Abstract] OR "exercise trainings"[Title/Abstract] OR "training exercise"[Title/Abstract] OR (("education"[MeSH Subheading] OR "education"[All Fields] OR "Training"[All Fields] OR "education"[MeSH Terms] OR "train"[All Fields] OR "train s"[All Fields] OR "trained"[All Fields] OR "training s"[All Fields] OR "Trainings"[All Fields] OR "trains"[All Fields]) AND "Exercise"[Title/Abstract]) OR "physical activity"[Title/Abstract] OR "activities physical"[Title/Abstract] OR "activity physical"[Title/Abstract] OR "physical activities"[Title/Abstract])) AND ("Intelligence"[MeSH Terms] OR "fluid intelligence"[Title/Abstract] OR "crystallized intelligence"[Title/Abstract]) AND (("child"[MeSH Terms] AND "Children"[Title/Abstract]) OR "Adolescent"[MeSH Terms] OR ("Adolescents"[Title/Abstract] OR "Adolescence"[Title/Abstract] OR "adolescents female"[Title/Abstract] OR "adolescent female"[Title/Abstract] OR "female adolescent"[Title/Abstract] OR "female adolescents"[Title/Abstract] OR "adolescents male"[Title/Abstract] OR "adolescent male"[Title/Abstract] OR "male adolescent"[Title/Abstract] OR "male adolescents"[Title/Abstract] OR "Youth"[Title/Abstract] OR "Youths"[Title/Abstract] OR "Teens"[Title/Abstract] OR "Teen"[Title/Abstract] OR "Teenagers"[Title/Abstract] OR "Teenager"[Title/Abstract]))) AND (clinicaltrial[Filter] OR randomizedcontrolledtrial[Filter]) | 294 |
| 12 | ("Exercise"[MeSH Terms] OR ("Exercises"[Title/Abstract] OR "exercise physical"[Title/Abstract] OR "exercises physical"[Title/Abstract] OR "physical exercise"[Title/Abstract] OR "physical exercises"[Title/Abstract] OR "exercise aerobic"[Title/Abstract] OR "aerobic exercise"[Title/Abstract] OR "aerobic exercises"[Title/Abstract] OR "exercises aerobic"[Title/Abstract] OR "exercise isometric"[Title/Abstract] OR "exercises isometric"[Title/Abstract] OR "isometric exercises"[Title/Abstract] OR "isometric exercise"[Title/Abstract] OR "exercise training"[Title/Abstract] OR "exercise trainings"[Title/Abstract] OR "training exercise"[Title/Abstract] OR (("education"[MeSH Subheading] OR "education"[All Fields] OR "Training"[All Fields] OR "education"[MeSH Terms] OR "train"[All Fields] OR "train s"[All Fields] OR "trained"[All Fields] OR "training s"[All Fields] OR "Trainings"[All Fields] OR "trains"[All Fields]) AND "Exercise"[Title/Abstract]) OR "physical activity"[Title/Abstract] OR "activities physical"[Title/Abstract] OR "activity physical"[Title/Abstract] OR "physical activities"[Title/Abstract])) AND ("Intelligence"[MeSH Terms] OR "fluid intelligence"[Title/Abstract] OR "crystallized intelligence"[Title/Abstract]) AND (("child"[MeSH Terms] AND "Children"[Title/Abstract]) OR "Adolescent"[MeSH Terms] OR ("Adolescents"[Title/Abstract] OR "Adolescence"[Title/Abstract] OR "adolescents female"[Title/Abstract] OR "adolescent female"[Title/Abstract] OR "female adolescent"[Title/Abstract] OR "female adolescents"[Title/Abstract] OR "adolescents male"[Title/Abstract] OR "adolescent male"[Title/Abstract] OR "male adolescent"[Title/Abstract] OR "male adolescents"[Title/Abstract] OR "Youth"[Title/Abstract] OR "Youths"[Title/Abstract] OR "Teens"[Title/Abstract] OR "Teen"[Title/Abstract] OR "Teenagers"[Title/Abstract] OR "Teenager"[Title/Abstract])) | 1,794 |
| 11 | "Exercise"[MeSH Terms] OR ("Exercises"[Title/Abstract] OR "exercise physical"[Title/Abstract] OR "exercises physical"[Title/Abstract] OR "physical exercise"[Title/Abstract] OR "physical exercises"[Title/Abstract] OR "exercise aerobic"[Title/Abstract] OR "aerobic exercise"[Title/Abstract] OR "aerobic exercises"[Title/Abstract] OR "exercises aerobic"[Title/Abstract] OR "exercise isometric"[Title/Abstract] OR "exercises isometric"[Title/Abstract] OR "isometric exercises"[Title/Abstract] OR "isometric exercise"[Title/Abstract] OR "exercise training"[Title/Abstract] OR "exercise trainings"[Title/Abstract] OR "training exercise"[Title/Abstract] OR (("education"[MeSH Subheading] OR "education"[All Fields] OR "Training"[All Fields] OR "education"[MeSH Terms] OR "train"[All Fields] OR "train s"[All Fields] OR "trained"[All Fields] OR "training s"[All Fields] OR "Trainings"[All Fields] OR "trains"[All Fields]) AND "Exercise"[Title/Abstract]) OR "physical activity"[Title/Abstract] OR "activities physical"[Title/Abstract] OR "activity physical"[Title/Abstract] OR "physical activities"[Title/Abstract]) | 476,250 |
| 10 | "Intelligence"[MeSH Terms] OR "fluid intelligence"[Title/Abstract] OR "crystallized intelligence"[Title/Abstract] | 138,644 |
| 9 | ("child"[MeSH Terms] AND "Children"[Title/Abstract]) OR "Adolescent"[MeSH Terms] OR ("Adolescents"[Title/Abstract] OR "Adolescence"[Title/Abstract] OR "adolescents female"[Title/Abstract] OR "adolescent female"[Title/Abstract] OR "female adolescent"[Title/Abstract] OR "female adolescents"[Title/Abstract] OR "adolescents male"[Title/Abstract] OR "adolescent male"[Title/Abstract] OR "male adolescent"[Title/Abstract] OR "male adolescents"[Title/Abstract] OR "Youth"[Title/Abstract] OR "Youths"[Title/Abstract] OR "Teens"[Title/Abstract] OR "Teen"[Title/Abstract] OR "Teenagers"[Title/Abstract] OR "Teenager"[Title/Abstract]) | 2,992,206 |
| 8 | "Exercises"[Title/Abstract] OR "exercise physical"[Title/Abstract] OR "exercises physical"[Title/Abstract] OR "physical exercise"[Title/Abstract] OR "physical exercises"[Title/Abstract] OR "exercise aerobic"[Title/Abstract] OR "aerobic exercise"[Title/Abstract] OR "aerobic exercises"[Title/Abstract] OR "exercises aerobic"[Title/Abstract] OR "exercise isometric"[Title/Abstract] OR "exercises isometric"[Title/Abstract] OR "isometric exercises"[Title/Abstract] OR "isometric exercise"[Title/Abstract] OR "exercise training"[Title/Abstract] OR "exercise trainings"[Title/Abstract] OR "training exercise"[Title/Abstract] OR (("education"[MeSH Subheading] OR "education"[All Fields] OR "Training"[All Fields] OR "education"[MeSH Terms] OR "train"[All Fields] OR "train s"[All Fields] OR "trained"[All Fields] OR "training s"[All Fields] OR "Trainings"[All Fields] OR "trains"[All Fields]) AND "Exercise"[Title/Abstract]) OR "physical activity"[Title/Abstract] OR "activities physical"[Title/Abstract] OR "activity physical"[Title/Abstract] OR "physical activities"[Title/Abstract] | 336,820 |
| 7 | "exercise"[MeSH Terms] | 269,742 |
| 6 | "crystallized intelligence"[Title/Abstract] | 356 |
| 5 | "fluid intelligence"[Title/Abstract] | 1,558 |
| 4 | "intelligence"[MeSH Terms] | 137,658 |
| 3 | "Adolescents"[Title/Abstract] OR "Adolescence"[Title/Abstract] OR "adolescents female"[Title/Abstract] OR "adolescent female"[Title/Abstract] OR "female adolescent"[Title/Abstract] OR "female adolescents"[Title/Abstract] OR "adolescents male"[Title/Abstract] OR "adolescent male"[Title/Abstract] OR "male adolescent"[Title/Abstract] OR "male adolescents"[Title/Abstract] OR "Youth"[Title/Abstract] OR "Youths"[Title/Abstract] OR "Teens"[Title/Abstract] OR "Teen"[Title/Abstract] OR "Teenagers"[Title/Abstract] OR "Teenager"[Title/Abstract] | 427,315 |
| 2 | "adolescent"[MeSH Terms] | 2,318,259 |
| 1 | "child"[MeSH Terms] AND "Children"[Title/Abstract] | 977,041 |

## Database: Embase <1974 to April 1 2025>

***Search Strategy:***

| No. | Query | Results |
| --- | --- | --- |
| #1 | ('child'/exp OR 'child':ti,ab OR 'children':ti,ab OR 'adolescent'/exp OR 'adolescent':ti,ab OR 'teenager':ti,ab) AND ('exercise'/exp OR 'biometric exercise':ti,ab OR 'effort':ti,ab OR 'exercise':ti,ab OR 'exercise capacity':ti,ab OR 'exercise performance':ti,ab OR 'exercise training':ti,ab OR 'exertion':ti,ab OR 'fitness training':ti,ab OR 'fitness workout':ti,ab OR 'physical conditioning, human':ti,ab OR 'physical effort':ti,ab OR 'physical exercise':ti,ab OR 'physical exertion':ti,ab OR 'physical work-out':ti,ab OR 'physical workout':ti,ab OR 'physical activity'/exp OR 'activity, physical' OR 'physical activity') AND ('intelligence'/exp OR 'intellectual ability' OR 'intellectual capability' OR 'intellectuality' OR 'intelligence' OR 'intelligence model' OR 'verbal intelligence') AND ('randomized controlled trial'/exp OR 'controlled trial, randomized' OR 'randomised controlled study' OR 'randomised controlled trial' OR 'randomized controlled study' OR 'randomized controlled trial' OR 'trial, randomized controlled') | 166 |

## Database: Cochrane

***Search Strategy:***

| ID | Search | Hits |
| --- | --- | --- |
| #1 | MeSH descriptor: [Exercise] explode all trees | 39577 |
| #2 | MeSH descriptor: [Intelligence] explode all trees | 8701 |
| #3 | MeSH descriptor: [Child] explode all trees | 81702 |
| #4 | MeSH descriptor: [Adolescent] explode all trees | 136093 |
| #5 | #3 OR #4 | 178952 |
| #6 | #1 AND #2 AND #5 | 236 |

## Database: Web of Science <1965 to April 1 2025>

***Search Strategy:***

| # | Search Query | Results |
| --- | --- | --- |
| 1 | TS=("Exercises" OR "exercise physical" OR "exercises physical" OR "physical exercise" OR "physical exercises" OR "exercise aerobic" OR "aerobic exercise" OR "aerobic exercises" OR "exercises aerobic" OR "exercise isometric" OR "exercises isometric" OR "isometric exercises" OR "isometric exercise" OR "exercise training" OR "exercise trainings" OR "training exercise" OR (("education"[MeSH Subheading] OR "education" OR "Training" OR "education" OR "train" OR "train s" OR "trained" OR "training s" OR "Trainings" OR "trains") AND "Exercise") OR "physical activity" OR "activities physical" OR "activity physical" OR "physical activities") and Preprint Citation Index (Exclude – Database) | 804340 |
| 2 | TS=("Adolescents" OR "Adolescence" OR "adolescents female" OR "adolescent female" OR "female adolescent" OR "female adolescents" OR "adolescents male" OR "adolescent male" OR "male adolescent" OR "male adolescents" OR "Youth" OR "Youths" OR "Teens" OR "Teen" OR "Teenagers" OR "Teenager") and Preprint Citation Index (Exclude – Database) | 1048483 |
| 3 | TS=("Intelligence" OR "fluid intelligence" OR "crystallized intelligence") and Preprint Citation Index (Exclude – Database) | 2206547 |
| 4 | TS=(“randomized controlled trial*” or “controlled clinical trial” or “random*” or “clinical trial*” or randomly or trial or “clinical trial” or “randomized controlled trial*” or “cross-over studies” or clinic*) and Preprint Citation Index (Exclude – Database) | 19562251 |
| 5 | #1 AND #2 AND #3 AND #4 and Preprint Citation Index (Exclude – Database) | 207 |

# Supplementary 2 Definitions and abbreviations of physical activity in the included literature

| Abbreviation | Full Name | Definitions |
| --- | --- | --- |
| AE | Aerobic Exercise | Aerobic exercise is performed by repeating sequences of light-to-moderate intensity activities for extended periods. e.g., walking, bicycle, and treadmill training etc. |
| BT | Balance training | Balance training refers to exercises and activities designed to improve an individual's ability to maintain stability and control their body’s position, both during static (non-moving) and dynamic (moving) conditions. The goal of balance training is to enhance the function of the body's proprioceptive and vestibular systems, which work together to detect changes in body position and maintain equilibrium. This training helps improve coordination, strength, and agility, and is often used to prevent falls, enhance athletic performance, and promote overall physical stability. |
| DTBT | Dual task balance training | Dual-task balance training refers to a type of exercise program that involves performing two tasks simultaneously—one of which is focused on maintaining balance while the other involves a cognitive or physical task. The purpose of dual-task balance training is to improve both physical stability and cognitive function by challenging the brain and body to work together in a coordinated manner. This approach is especially beneficial in training for real-world situations where individuals need to maintain balance while also performing other tasks, such as walking while talking, carrying objects, or navigating complex environments. |
| Mul_C | Multi-component exercise | Two or more of the above specific types of exercise training (if it is only part of warm-up or relaxation, it is not considered as multi-mode) |
| PMT | Perceptual motor training | Perceptual motor training refers to a type of exercise and training regimen designed to enhance the integration of sensory perception (such as sight, sound, touch, and proprioception) with motor actions (movements or physical responses). The goal of perceptual motor training is to improve the coordination between the brain’s sensory processing systems and the body’s motor responses, helping individuals to react more quickly, accurately, and efficiently to their environment. This type of training is often used to enhance skill development, improve reaction times, and facilitate learning new physical tasks or improving existing motor skills. |
| Yoga | NA | Mainly a series of methods for self-cultivation, including body-adjusting asanas (refer to yoga asana collection), breathing-adjusting breathing methods, and mind-adjusting meditation, etc., to achieve the unity of body and mind. |
| CON | Control group | No intervention, maintained their regular classroom schedules, or wait list. |

# Supplementary 3: Risk of Bias

## Table 3 The risk of bias assessment for the individual included studies

| **Study** | **Randomization process** | **Deviations from intended interventions** | **Missing outcome data** | **Measurement of the outcome** | **Selection of the reported result** | **Overall risk of bias** |
| --- | --- | --- | --- | --- | --- | --- |
| Ardoy et al. (2014) | Low | Low | Some concerns | Low | Low | Some concerns |
| Chaddock-Heyman et al. (2013) | Low | Low | Low | Low | Low | Low |
| Corder (1966) | Low | Low | Low | Low | Low | Low |
| Fisher (1971) | Low | Low | Low | Low | Low | Low |
| Ortega et al. (2022) | Low | Low | Low | Low | Low | Low |
| Hirata (2023) | Some concerns | Low | Some concerns | Low | Low | Some concerns |
| Atak et al. (2023) | Some concerns | Low | Some concerns | Low | Low | Some concerns |
| Parajuli et al. (2022) | Some concerns | Low | Low | Low | Low | Some concerns |
| Reed et al. (2010) | Some concerns | Low | Low | High | Low | High |
| Sánchez-López et al. (2019) | Some concerns | Low | Low | Low | High | High |
| Uma et al. (1989) | Low | Low | Some concerns | Low | Low | Some concerns |
| Chaya et al. (2012) | Low | Low | Low | Low | Low | Low |
| Rymarczyk et al. (2024) | Low | Low | Low | Low | Low | Low |
| Jia et al. (2021) | Low | Low | Low | Low | Low | Low |
| Fedewa et al. (2015) | Low | Low | Low | Low | Low | Low |

# Supplementary 4: Pairwise meta-analysis

# Supplementary 5: Details of SIDE splitting results

Table 5.1: Details of SIDE splitting result for general intelligence

| **Comparison** | **k** | **prop** | **nma** | **direct** | **indir.** | **Diff** | **z** | **p-value** |
| --- | --- | --- | --- | --- | --- | --- | --- | --- |
| AE vs BT | 0 | 0 | 0.2204 | NA | 0.2204 | NA | NA | NA |
| AE vs CON | 0 | 0 | 0.3629 | NA | 0.3629 | NA | NA | NA |
| AE vs DTBT | 0 | 0 | -0.5706 | NA | -0.5706 | NA | NA | NA |
| AE vs Mul_C | 1 | 1 | -0.2393 | -0.2393 | NA | NA | NA | NA |
| AE vs PMT | 0 | 0 | -0.0208 | NA | -0.0208 | NA | NA | NA |
| AE vs Yoga | 0 | 0 | -0.2122 | NA | -0.2122 | NA | NA | NA |
| BT vs CON | 1 | 1 | 0.1425 | 0.1425 | NA | NA | NA | NA |
| BT vs DTBT | 1 | 1 | -0.7909 | -0.7909 | NA | NA | NA | NA |
| BT vs Mul_C | 0 | 0 | -0.4596 | NA | -0.4596 | NA | NA | NA |
| BT vs PMT | 0 | 0 | -0.2411 | NA | -0.2411 | NA | NA | NA |
| BT vs Yoga | 0 | 0 | -0.4326 | NA | -0.4326 | NA | NA | NA |
| DTBT vs CON | 1 | 1 | 0.9334 | 0.9334 | NA | NA | NA | NA |
| Mul_C vs CON | 6 | 0.89 | 0.6021 | 0.6156 | 0.496 | 0.1197 | 0.4 | 0.6898 |
| PMT vs CON | 1 | 1 | 0.3837 | 0.3837 | NA | NA | NA | NA |
| Yoga vs CON | 1 | 0.34 | 0.5751 | 0.4965 | 0.6162 | -0.1197 | -0.4 | 0.6898 |
| DTBT vs Mul_C | 0 | 0 | 0.3313 | NA | 0.3313 | NA | NA | NA |
| DTBT vs PMT | 0 | 0 | 0.5498 | NA | 0.5498 | NA | NA | NA |
| DTBT vs Yoga | 0 | 0 | 0.3584 | NA | 0.3584 | NA | NA | NA |
| Mul_C vs PMT | 0 | 0 | 0.2185 | NA | 0.2185 | NA | NA | NA |
| Mul_C vs Yoga | 1 | 0.77 | 0.0271 | -0.0005 | 0.1192 | -0.1197 | -0.4 | 0.6898 |
| PMT vs Yoga | 0 | 0 | -0.1914 | NA | -0.1914 | NA | NA | NA |

NA not available

k - Number of studies providing direct evidence

prop - Direct evidence proportion

nma - Estimated treatment effect (SMD) in network meta-analysis

direct - Estimated treatment effect (SMD) derived from direct evidence

indir. - Estimated treatment effect (SMD) derived from indirect evidence

Diff - Difference between direct and indirect treatment estimates

z - z-value of test for disagreement (direct versus indirect)

p-value - p-value of test for disagreement (direct versus indirect)

Table 5.2: Details of SIDE splitting result for fluid intelligence

| **Comparison** | **k** | **prop** | **nma** | **direct** | **indir.** | **Diff** | **z** | **p-value** |
| --- | --- | --- | --- | --- | --- | --- | --- | --- |
| AE vs BT | 0 | 0 | -0.133 | NA | -0.133 | NA | NA | NA |
| AE vs CON | 2 | 0.92 | 0.1312 | 0.1301 | 0.1441 | -0.014 | -0.04 | 0.9669 |
| AE vs DTBT | 0 | 0 | -0.8424 | NA | -0.8424 | NA | NA | NA |
| AE vs Mul_C | 1 | 0.1 | -0.3681 | -0.3556 | -0.3695 | 0.014 | 0.04 | 0.9669 |
| AE vs PMT | 0 | 0 | -0.3118 | NA | -0.3118 | NA | NA | NA |
| AE vs Yoga | 0 | 0 | -0.4078 | NA | -0.4078 | NA | NA | NA |
| BT vs CON | 1 | 1 | 0.2643 | 0.2643 | NA | NA | NA | NA |
| BT vs DTBT | 1 | 1 | -0.7094 | -0.7094 | NA | NA | NA | NA |
| BT vs Mul_C | 0 | 0 | -0.2351 | NA | -0.2351 | NA | NA | NA |
| BT vs PMT | 0 | 0 | -0.1788 | NA | -0.1788 | NA | NA | NA |
| BT vs Yoga | 0 | 0 | -0.2747 | NA | -0.2747 | NA | NA | NA |
| DTBT vs CON | 1 | 1 | 0.9736 | 0.9736 | NA | NA | NA | NA |
| Mul_C vs CON | 12 | 0.98 | 0.4994 | 0.4996 | 0.4857 | 0.014 | 0.04 | 0.9669 |
| PMT vs CON | 1 | 1 | 0.443 | 0.443 | NA | NA | NA | NA |
| Yoga vs CON | 0 | 0 | 0.539 | NA | 0.539 | NA | NA | NA |
| DTBT vs Mul_C | 0 | 0 | 0.4743 | NA | 0.4743 | NA | NA | NA |
| DTBT vs PMT | 0 | 0 | 0.5306 | NA | 0.5306 | NA | NA | NA |
| DTBT vs Yoga | 0 | 0 | 0.4346 | NA | 0.4346 | NA | NA | NA |
| Mul_C vs PMT | 0 | 0 | 0.0563 | NA | 0.0563 | NA | NA | NA |
| Mul_C vs Yoga | 2 | 1 | -0.0396 | -0.0396 | NA | NA | NA | NA |
| PMT vs Yoga | 0 | 0 | -0.0959 | NA | -0.0959 | NA | NA | NA |

NA not available

k - Number of studies providing direct evidence

prop - Direct evidence proportion

nma - Estimated treatment effect (SMD) in network meta-analysis

direct - Estimated treatment effect (SMD) derived from direct evidence

indir. - Estimated treatment effect (SMD) derived from indirect evidence

Diff - Difference between direct and indirect treatment estimates

z - z-value of test for disagreement (direct versus indirect)

p-value - p-value of test for disagreement (direct versus indirect)

Table 5.3: Details of SIDE splitting result for crystallized intelligence

| **Comparison** | **k** | **prop** | **nma** | **direct** | **indir.** | **Diff** | **z** | **p-value** |
| --- | --- | --- | --- | --- | --- | --- | --- | --- |
| AE vs BT | 0 | 0 | 0.4902 | NA | 0.4902 | NA | NA | NA |
| AE vs CON | 0 | 0 | 0.579 | NA | 0.579 | NA | NA | NA |
| AE vs DTBT | 0 | 0 | -0.4711 | NA | -0.4711 | NA | NA | NA |
| AE vs Mul_C | 1 | 1 | -0.1255 | -0.1255 | NA | NA | NA | NA |
| AE vs PMT | 0 | 0 | 0.43 | NA | 0.43 | NA | NA | NA |
| AE vs Yoga | 0 | 0 | -0.0454 | NA | -0.0454 | NA | NA | NA |
| BT vs CON | 1 | 1 | 0.0888 | 0.0888 | NA | NA | NA | NA |
| BT vs DTBT | 1 | 1 | -0.9613 | -0.9613 | NA | NA | NA | NA |
| BT vs Mul_C | 0 | 0 | -0.6157 | NA | -0.6157 | NA | NA | NA |
| BT vs PMT | 0 | 0 | -0.0602 | NA | -0.0602 | NA | NA | NA |
| BT vs Yoga | 0 | 0 | -0.5355 | NA | -0.5355 | NA | NA | NA |
| DTBT vs CON | 1 | 1 | 1.0501 | 1.0501 | NA | NA | NA | NA |
| Mul_C vs CON | 6 | 1 | 0.7045 | 0.7045 | NA | NA | NA | NA |
| PMT vs CON | 1 | 1 | 0.149 | 0.149 | NA | NA | NA | NA |
| Yoga vs CON | 0 | 0 | 0.6243 | NA | 0.6243 | NA | NA | NA |
| DTBT vs Mul_C | 0 | 0 | 0.3456 | NA | 0.3456 | NA | NA | NA |
| DTBT vs PMT | 0 | 0 | 0.9011 | NA | 0.9011 | NA | NA | NA |
| DTBT vs Yoga | 0 | 0 | 0.4257 | NA | 0.4257 | NA | NA | NA |
| Mul_C vs PMT | 0 | 0 | 0.5555 | NA | 0.5555 | NA | NA | NA |
| Mul_C vs Yoga | 1 | 1 | 0.0801 | 0.0801 | NA | NA | NA | NA |
| PMT vs Yoga | 0 | 0 | -0.4753 | NA | -0.4753 | NA | NA | NA |

NA not available

k - Number of studies providing direct evidence

prop - Direct evidence proportion

nma - Estimated treatment effect (SMD) in network meta-analysis

direct - Estimated treatment effect (SMD) derived from direct evidence

indir. - Estimated treatment effect (SMD) derived from indirect evidence

Diff - Difference between direct and indirect treatment estimates

z - z-value of test for disagreement (direct versus indirect)

p-value - p-value of test for disagreement (direct versus indirect)

# Supplementary 6: Publication bias

# Supplementary 7:The results of network meta-analysis

Figure 7.1: Network plot of general intelligence

Table 7.1 The league table of general intelligence

| **DTBT**  **(0.89)** | NA | NA | NA | NA | **0.79 ( 0.05; 1.53)** | **0.93 ( 0.19; 1.68)** |
| --- | --- | --- | --- | --- | --- | --- |
| 0.33 (-0.44; 1.10) | **Mul_C**  **(0.69)** | -0.00 (-0.28; 0.28) | NA | 0.24 (-0.38; 0.86) | NA | **0.62 ( 0.42; 0.81)** |
| 0.36 (-0.44; 1.15) | 0.03 (-0.22; 0.27) | **Yoga**  **(0.65)** | NA | NA | NA | **0.50 ( 0.02; 0.97)** |
| 0.55 (-0.47; 1.57) | 0.22 (-0.51; 0.94) | 0.19 (-0.56; 0.95) | **PMT**  **(0.46)** | NA | NA | 0.38 (-0.32; 1.08) |
| 0.57 (-0.42; 1.56) | 0.24 (-0.38; 0.86) | 0.21 (-0.46; 0.88) | 0.02 (-0.93; 0.98) | **AE**  **(0.44)** | NA | NA |
| **0.79 ( 0.05; 1.53)** | 0.46 (-0.28; 1.20) | 0.43 (-0.34; 1.20) | 0.24 (-0.76; 1.24) | 0.22 (-0.75; 1.19) | **BT**  **(0.26)** | 0.14 (-0.57; 0.86) |
| **0.93 ( 0.19; 1.68)** | **0.60 ( 0.42; 0.79)** | **0.58 ( 0.30; 0.85)** | 0.38 (-0.32; 1.08) | 0.36 (-0.29; 1.01) | 0.14 (-0.57; 0.86) | **CON**  **(0.11)** |

All results are presented in the form of SMD (95% CrI). Treatment types are ranked according to the SUCRA for physical activity with the best from left to right. The results of the network meta-analysis are showed in the lower left part, and results from pairwise comparisons in the upper right half (if available). Cells shown in bold indicate significant results. NA not available, SMD standardized mean difference, CrI Credible Interval.

Figure 7.2: Network plot of fluid intelligence

Table 7.2 The league table of fluid intelligence

| **DTBT**  **(0.92)** | NA | NA | NA | 0.71 (-0.02; 1.44) | NA | **0.97 ( 0.23; 1.72)** |
| --- | --- | --- | --- | --- | --- | --- |
| 0.43 (-0.35; 1.22) | **Yoga**  **(0.69)** | 0.04 (-0.20; 0.27) | NA | NA | NA | NA |
| 0.47 (-0.28; 1.23) | 0.04 (-0.20; 0.27) | **Mul_C**  **(0.63)** | NA | NA | 0.36 (-0.27; 0.98) | **0.50 ( 0.41; 0.59)** |
| 0.53 (-0.50; 1.56) | 0.10 (-0.65; 0.84) | 0.06 (-0.65; 0.77) | **PMT**  **(0.55)** | NA | NA | 0.44 (-0.26; 1.15) |
| 0.71 (-0.02; 1.44) | 0.27 (-0.49; 1.04) | 0.24 (-0.49; 0.96) | 0.18 (-0.83; 1.18) | **BT**  **(0.38)** | NA | 0.26 (-0.45; 0.98) |
| **0.84 ( 0.07; 1.61)** | **0.41 ( 0.10; 0.71)** | 0.37 ( 0.17; 0.57) | 0.31 (-0.41; 1.04) | 0.13 (-0.61; 0.87) | **AE**  **(0.25)** | 0.13 (-0.06; 0.32) |
| **0.97 ( 0.23; 1.72)** | **0.54 ( 0.29; 0.79)** | **0.50 ( 0.41; 0.59)** | 0.44 (-0.26; 1.15) | 0.26 (-0.45; 0.98) | 0.13 (-0.05; 0.31) | **CON**  **(0.07)** |

All results are presented in the form of SMD (95% CrI). Treatment types are ranked according to the SUCRA for physical activity with the best from left to right. The results of the network meta-analysis are showed in the lower left part, and results from pairwise comparisons in the upper right half (if available). Cells shown in bold indicate significant results. NA not available, SMD standardized mean difference, CrI Credible Interval.

Figure 7.3: Network plot of crystallized intelligence

Table 7.3 The league table of crystallized intelligence

| **DTBT**  **(0.90)** | NA | NA | NA | NA | **0.96 ( 0.20; 1.72)** | **1.05 ( 0.28; 1.82)** |
| --- | --- | --- | --- | --- | --- | --- |
| 0.35 (-0.47; 1.16) | **Mul_C**  **(0.73)** | 0.08 (-0.24; 0.40) | 0.13 (-0.51; 0.76) | NA | NA | **0.70 ( 0.44; 0.97)** |
| 0.43 (-0.45; 1.30) | 0.08 (-0.24; 0.40) | **Yoga**  **(0.63)** | NA | NA | NA | NA |
| 0.47 (-0.56; 1.50) | 0.13 (-0.51; 0.76) | 0.05 (-0.67; 0.76) | **AE**  **(0.59)** | NA | NA | NA |
| 0.90 (-0.14; 1.95) | 0.56 (-0.20; 1.31) | 0.48 (-0.35; 1.30) | 0.43 (-0.56; 1.42) | **PMT**  **(0.28)** | NA | 0.15 (-0.56; 0.86) |
| **0.96 ( 0.20; 1.72)** | 0.62 (-0.16; 1.39) | 0.54 (-0.30; 1.38) | 0.49 (-0.51; 1.50) | 0.06 (-0.96; 1.08) | **BT**  **(0.23)** | 0.09 (-0.64; 0.82) |
| **1.05 ( 0.28; 1.82)** | **0.70 ( 0.44; 0.97)** | **0.62 ( 0.21; 1.04)** | 0.58 (-0.11; 1.27) | 0.15 (-0.56; 0.86) | 0.09 (-0.64; 0.82) | **CON**  **(0.13)** |

All results are presented in the form of SMD (95% CrI). Treatment types are ranked according to the SUCRA for physical activity with the best from left to right. The results of the network meta-analysis are showed in the lower left part, and results from pairwise comparisons in the upper right half (if available). Cells shown in bold indicate significant results. NA not available, SMD standardized mean difference, CrI Credible Interval.

Supplementary 8: Non-linera and linear functions and models fit comparison

Table 8.1-3 shows the fit indices from each of the models fitted. DIC = Deviance Information Criterion; SD = Between-study Standard Deviation; pD: Number of estimated parameters; NA = Not Applicable. The SD is presented as the main value.

**Table 8.1** Models fit comparison (Effect of single physical activity time on intelligence)

| Model | DIC | SD | Deviance | Residual deviance | pD |
| --- | --- | --- | --- | --- | --- |
| Emax  (common treatment effects) | 425.5 | NA | 385.035 | 92.935 | 41.1 |
| Emax  (RANDOM treatment effects) | 427.3 | 0.082 | 380.574 | 88.473 | 46.9 |
| Linear  (common treatment effects) | 427.1 | NA | 386.764 | 94.663 | 41.0 |
| Linear  (random treatment effects) | 431.4 | 0.076 | 382.405 | 90.304 | 49.1 |
| EXponential  (common treatment effects) | 432.9 | NA | 390.376 | 98.275 | 42.7 |
| EXponential  (RANDOM treatment effects) | 427.6 | 0.169 | 375.062 | 82.961 | 52.9 |
| Restricted cubic spline  (common treatment effects; 3 knots) | 426.4 | NA | 384.663 | 92.562 | 42.2 |
| Restricted cubic spline  (random treatment effects; 3 knots) | 426.6 | 0.096 | 379.193 | 87.092 | 47.6 |

**Table 8.2** Models fit comparison (Effects of weekly physical activity frequency on intelligence)

| Model | DIC | SD | Deviance | Residual deviance | pD |
| --- | --- | --- | --- | --- | --- |
| Emax  (common treatment effects) | 441.5 | NA | 401.157 | 109.056 | 40.9 |
| Emax  (RANDOM treatment effects) | 432.3 | 0.235 | 373.292 | 81.192 | 59.4 |
| Linear  (common treatment effects) | 466.4 | NA | 425.927 | 133.826 | 41.1 |
| Linear  (random treatment effects) | 432.0 | 0.243 | 373.509 | 81.408 | 58.9 |
| EXponential  (common treatment effects) | 438.6 | NA | 398.366 | 106.265 | 41.0 |
| EXponential  (RANDOM treatment effects) | 428.8 | 0.173 | 374.599 | 82.498 | 54.4 |
| Restricted cubic spline  (common treatment effects; 3 knots) | 434.7 | NA | 393.249 | 101.148 | 42.1 |
| Restricted cubic spline  (random treatment effects; 3 knots) | 421.9 | 0.160 | 371.501 | 80.400 | 54.9 |

**Table 8.3** Models fit comparison (Effects of weekly physical activity minutes on intelligence)

| Model | DIC | SD | Deviance | Residual deviance | pD |
| --- | --- | --- | --- | --- | --- |
| Emax  (common treatment effects) | 434.6 | NA | 393.655 | 101.554 | 41.7 |
| Emax  (RANDOM treatment effects) | 428.3 | 0.154 | 376.865 | 84.764 | 51.7 |
| Linear  (common treatment effects) | 449.6 | NA | 409.358 | 117.257 | 40.9 |
| Linear  (random treatment effects) | 433.8 | 0.204 | 377.839 | 85.739 | 56.6 |
| EXponential  (common treatment effects) | 439.1 | NA | 398.589 | 106.488 | 41.2 |
| EXponential  (RANDOM treatment effects) | 429.5 | 0.170 | 375.105 | 83.004 | 54.6 |
| Restricted cubic spline  (common treatment effects; 3 knots) | 434.2 | NA | 392.753 | 100.652 | 42.3 |
| Restricted cubic spline  (random treatment effects; 3 knots) | 420.1 | 0.144 | 378.403 | 86.302 | 51.6 |

**Table 8.4.** Models fit comparison (Effects of physical activity periods on intelligence)

| Model | DIC | SD | Deviance | Residual deviance | pD |
| --- | --- | --- | --- | --- | --- |
| Emax  (common treatment effects) | 441.3 | NA | 400.984 | 108.883 | 41.0 |
| Emax  (RANDOM treatment effects) | 430.6 | 0.203 | 374.721 | 82.620 | 56.2 |
| Linear  (common treatment effects) | 484.5 | NA | 443.875 | 151.774 | 41.3 |
| Linear  (random treatment effects) | 438.4 | 0.301 | 375.756 | 83.655 | 63.1 |
| EXponential  (common treatment effects) | 438.5 | NA | 398.419 | 106.318 | 40.6 |
| EXponential  (RANDOM treatment effects) | 435.0 | 0.275 | 374.803 | 82.702 | 53.7 |
| Restricted cubic spline  (common treatment effects; 3 knots) | 440.4 | NA | 398.773 | 106.672 | 42.2 |
| Restricted cubic spline  (random treatment effects; 3 knots) | 432.1 | 0.180 | 376.969 | 84.868 | 55.7 |

Supplementary 9: Grading the evidence of the network meta-analysis using CINeMA

Based on the recommendations of the CINeMA online document (https://cinema.ispm.unibe.ch/), we judged whether each module needs to be downgraded according to the following criteria.

***With-study bias***

We classified the quality evaluation results of each included study into low-risk, some concern, and high-risk according to the standards recommended by Huhn, Nikolakopoulou ^1^. We selected the rule is average RoB. No need to downgrade when the result was “no concerns”, downgrade one level when “some concerns” and downgrade two level “major concerns”.

***Across-study bias (publication bias)***

In supplementary 5, we evaluated the outcomes of publication bias, and comparison-adjusted funnel plots for PA types vs. CON show no evidence of asymmetry. Therefore, the outcomes were deemed to have no publication bias.

***Indirectness***

Statistical consistency was evaluated using the design-by-treatment test ^2^ and by differentiating indirect from direct evidence (SIDE test) ^3^ via the R ‘netmeta’ package. In addition, we also use the leverage diagram to select the consistency model or the inconsistency model (**Supplementary File 4**). ***Imprecision***

The outcomes this network meta-analysis was continuous variable, and the effect size measure for continuous outcomes chooses the standaridized mean difference (SMD) of the change score (end-point minus baseline score).Therefore, for CON comparisons the clinically meaningful threshold was set at a SMD of higher or lower than 0. If the confidence interval crossed threshold, it will be downgraded by one leve.

***Heterogeneity***

For heterogeneity, we used the same threshold as the above clinically significant threshold and follow the recommendations automatically provided by CINeMA (https://cinema.ispm.unibe.ch/). No need to downgrade when the result was “no concerns”, downgrade one level when “some concerns” and downgrade two level when “major concerns”.

***Incoherence***

For incoherence, we will use global and local methods to test the inconsistency of the research results. For global inconsistency, we evaluated inconsistency statistically using the design-by-treatment test. In addition, we will assessment of local inconsistency by separating indirect from direct evidence (SIDE test) using the R netmeta package (**Supplementary 4**). No need to downgrade when p >0.1, downgrade one level when p was 0.05-0.1 and downgrade two level when p <0.05 .

***Summarising judgments across the 6 domains***

Τhe final output of CINeMA is a table with the level of concern for each of the 6 domains. we choose to summarise judgments across domains using the 4 levels of confidence of the GRADE approach: very low, low, moderate, or high.^4^ Due to factors that may reduce the confidence in a treatment effect may affect more than 1 domain. Indirectness includes consideration of intransitivity, which is manifested as statistical incoherence in the data. Heterogeneity will increase the imprecision of treatment effect, and may be related to the variability of bias within the study or the existence of reporting bias. In addition, in the presence of heterogeneity, the ability to detect important discontinuities will be reduced.^5^ Therefore, the 6 CINeMA domains should be considered jointly rather than in isolation to avoid downgrading the overall level of confidence more than once for related concerns.

## 9.1 CINeMA for the effects of general intelligence

| **Comparison** | **Number of studies** | **Within-study bias** | **Reporting bias** | **Indirectness** | **Imprecision** | **Heterogeneity** | **Incoherence** | **Confidence rating** | **Reason(s) for downgrading** |
| --- | --- | --- | --- | --- | --- | --- | --- | --- | --- |
| AE:Mul_C | 1 | No concerns | Low risk | No concerns | Major concerns | No concerns | No concerns | Low | ["Imprecision"] |
| BT:CON | 1 | Some concerns | Low risk | No concerns | Major concerns | No concerns | No concerns | Very low | ["Within-study bias","Imprecision"] |
| BT:DTBT | 1 | Some concerns | Low risk | No concerns | No concerns | Major concerns | No concerns | Very low | ["Within-study bias","Heterogeneity"] |
| CON:DTBT | 1 | Some concerns | Low risk | No concerns | No concerns | Major concerns | No concerns | Very low | ["Within-study bias","Heterogeneity"] |
| CON:Mul_C | 6 | Some concerns | Low risk | No concerns | No concerns | No concerns | No concerns | Moderate | ["Within-study bias"] |
| CON:PMT | 1 | No concerns | Low risk | No concerns | Major concerns | No concerns | No concerns | Low | ["Imprecision"] |
| CON:Yoga | 1 | Some concerns | Low risk | No concerns | No concerns | No concerns | No concerns | Moderate | ["Within-study bias"] |
| Mul_C:Yoga | 1 | No concerns | Low risk | No concerns | Major concerns | No concerns | No concerns | Low | ["Imprecision"] |
| AE:BT | 0 | Some concerns | Low risk | No concerns | Major concerns | No concerns | No concerns | Very low | ["Within-study bias","Imprecision"] |
| AE:CON | 0 | Some concerns | Low risk | No concerns | Major concerns | No concerns | No concerns | Very low | ["Within-study bias","Imprecision"] |
| AE:DTBT | 0 | Some concerns | Low risk | No concerns | Major concerns | No concerns | No concerns | Very low | ["Within-study bias","Imprecision"] |
| AE:PMT | 0 | No concerns | Low risk | No concerns | Major concerns | No concerns | No concerns | Low | ["Imprecision"] |
| AE:Yoga | 0 | No concerns | Low risk | No concerns | Major concerns | No concerns | No concerns | Low | ["Imprecision"] |
| BT:Mul_C | 0 | Some concerns | Low risk | No concerns | Major concerns | No concerns | No concerns | Very low | ["Within-study bias","Imprecision"] |
| BT:PMT | 0 | Some concerns | Low risk | No concerns | Major concerns | No concerns | No concerns | Very low | ["Within-study bias","Imprecision"] |
| BT:Yoga | 0 | Some concerns | Low risk | No concerns | Major concerns | No concerns | No concerns | Very low | ["Within-study bias","Imprecision"] |
| DTBT:Mul_C | 0 | Some concerns | Low risk | No concerns | Major concerns | No concerns | No concerns | Very low | ["Within-study bias","Imprecision"] |
| DTBT:PMT | 0 | Some concerns | Low risk | No concerns | Major concerns | No concerns | No concerns | Very low | ["Within-study bias","Imprecision"] |
| DTBT:Yoga | 0 | Some concerns | Low risk | No concerns | Major concerns | No concerns | No concerns | Very low | ["Within-study bias","Imprecision"] |
| Mul_C:PMT | 0 | Some concerns | Low risk | No concerns | Major concerns | No concerns | No concerns | Very low | ["Within-study bias","Imprecision"] |
| PMT:Yoga | 0 | No concerns | Low risk | No concerns | Major concerns | No concerns | No concerns | Low | ["Imprecision"] |

## 9.2 CINeMA for the effects of fluid intelligence

| **Comparison** | **Number of studies** | **Within-study bias** | **Reporting bias** | **Indirectness** | **Imprecision** | **Heterogeneity** | **Incoherence** | **Confidence rating** | **Reason(s) for downgrading** |
| --- | --- | --- | --- | --- | --- | --- | --- | --- | --- |
| AE:CON | 2 | No concerns | Low risk | No concerns | Major concerns | No concerns | No concerns | Low | ["Imprecision"] |
| AE:Mul_C | 1 | No concerns | Low risk | No concerns | No concerns | No concerns | No concerns | High | [] |
| BT:CON | 1 | Some concerns | Low risk | No concerns | Major concerns | No concerns | No concerns | Very low | ["Within-study bias","Imprecision"] |
| BT:DTBT | 1 | Some concerns | Low risk | No concerns | Major concerns | No concerns | No concerns | Very low | ["Within-study bias","Imprecision"] |
| CON:DTBT | 1 | Some concerns | Low risk | No concerns | No concerns | No concerns | No concerns | Moderate | ["Within-study bias"] |
| CON:Mul_C | 12 | Some concerns | Low risk | No concerns | No concerns | No concerns | No concerns | Moderate | ["Within-study bias"] |
| CON:PMT | 1 | No concerns | Low risk | No concerns | Major concerns | No concerns | No concerns | Low | ["Imprecision"] |
| Mul_C:Yoga | 2 | No concerns | Low risk | No concerns | Major concerns | No concerns | No concerns | Low | ["Imprecision"] |
| AE:BT | 0 | Some concerns | Low risk | No concerns | Major concerns | No concerns | No concerns | Very low | ["Within-study bias","Imprecision"] |
| AE:DTBT | 0 | Some concerns | Low risk | No concerns | No concerns | Major concerns | No concerns | Very low | ["Within-study bias","Heterogeneity"] |
| AE:PMT | 0 | No concerns | Low risk | No concerns | Major concerns | No concerns | No concerns | Low | ["Imprecision"] |
| AE:Yoga | 0 | No concerns | Low risk | No concerns | No concerns | No concerns | No concerns | High | [] |
| BT:Mul_C | 0 | Some concerns | Low risk | No concerns | Major concerns | No concerns | No concerns | Very low | ["Within-study bias","Imprecision"] |
| BT:PMT | 0 | Some concerns | Low risk | No concerns | Major concerns | No concerns | No concerns | Very low | ["Within-study bias"] |
| BT:Yoga | 0 | Some concerns | Low risk | No concerns | Major concerns | No concerns | No concerns | Very low | ["Within-study bias"] |
| CON:Yoga | 0 | Some concerns | Low risk | No concerns | No concerns | No concerns | No concerns | Moderate | ["Within-study bias"] |
| DTBT:Mul_C | 0 | Some concerns | Low risk | No concerns | Major concerns | No concerns | No concerns | Very low | ["Within-study bias","Imprecision"] |
| DTBT:PMT | 0 | Some concerns | Low risk | No concerns | Major concerns | No concerns | No concerns | Very low | ["Within-study bias","Imprecision"] |
| DTBT:Yoga | 0 | Some concerns | Low risk | No concerns | Major concerns | No concerns | No concerns | Very low | ["Within-study bias","Imprecision"] |
| Mul_C:PMT | 0 | Some concerns | Low risk | No concerns | Major concerns | No concerns | No concerns | Very low | ["Within-study bias","Imprecision"] |
| PMT:Yoga | 0 | No concerns | Low risk | No concerns | Major concerns | No concerns | No concerns | Low | ["Imprecision"] |

## 9.3 CINeMA for the effects of crystallized intelligence

| **Comparison** | **Number of studies** | **Within-study bias** | **Reporting bias** | **Indirectness** | **Imprecision** | **Heterogeneity** | **Incoherence** | **Confidence rating** | **Reason(s) for downgrading** |
| --- | --- | --- | --- | --- | --- | --- | --- | --- | --- |
| AE:Mul_C | 1 | No concerns | Low risk | No concerns | Major concerns | No concerns | Major concerns | Low | ["Imprecision","Incoherence"] |
| BT:CON | 1 | Some concerns | Low risk | No concerns | Major concerns | No concerns | Major concerns | Very low | ["Within-study bias","Imprecision","Incoherence"] |
| BT:DTBT | 1 | Some concerns | Low risk | No concerns | No concerns | Major concerns | Major concerns | Very low | ["Within-study bias","Heterogeneity","Incoherence"] |
| CON:DTBT | 1 | Some concerns | Low risk | No concerns | No concerns | Major concerns | Major concerns | Very low | ["Within-study bias","Heterogeneity","Incoherence"] |
| CON:Mul_C | 6 | Some concerns | Low risk | No concerns | No concerns | No concerns | Major concerns | Very low | ["Within-study bias","Incoherence"] |
| CON:PMT | 1 | No concerns | Low risk | No concerns | Major concerns | No concerns | Major concerns | Low | ["Imprecision","Incoherence"] |
| Mul_C:Yoga | 1 | No concerns | Low risk | No concerns | Major concerns | No concerns | Major concerns | Low | ["Imprecision","Incoherence"] |
| AE:BT | 0 | Some concerns | Low risk | No concerns | Major concerns | No concerns | Major concerns | Very low | ["Within-study bias","Imprecision","Incoherence"] |
| AE:CON | 0 | No concerns | Low risk | No concerns | Major concerns | No concerns | Major concerns | Low | ["Imprecision","Incoherence"] |
| AE:DTBT | 0 | Some concerns | Low risk | No concerns | Major concerns | No concerns | Major concerns | Very low | ["Within-study bias","Imprecision","Incoherence"] |
| AE:PMT | 0 | No concerns | Low risk | No concerns | Major concerns | No concerns | Major concerns | Low | ["Imprecision","Incoherence"] |
| AE:Yoga | 0 | No concerns | Low risk | No concerns | Major concerns | No concerns | Major concerns | Low | ["Imprecision","Incoherence"] |
| BT:Mul_C | 0 | Some concerns | Low risk | No concerns | Major concerns | No concerns | Major concerns | Very low | ["Within-study bias","Imprecision","Incoherence"] |
| BT:PMT | 0 | Some concerns | Low risk | No concerns | Major concerns | No concerns | Major concerns | Very low | ["Within-study bias","Imprecision","Incoherence"] |
| BT:Yoga | 0 | Some concerns | Low risk | No concerns | Major concerns | No concerns | Major concerns | Very low | ["Within-study bias","Imprecision","Incoherence"] |
| CON:Yoga | 0 | No concerns | Low risk | No concerns | No concerns | Major concerns | Major concerns | Low | ["Heterogeneity","Incoherence"] |
| DTBT:Mul_C | 0 | Some concerns | Low risk | No concerns | Major concerns | No concerns | Major concerns | Very low | ["Within-study bias","Imprecision","Incoherence"] |
| DTBT:PMT | 0 | Some concerns | Low risk | No concerns | Major concerns | No concerns | Major concerns | Very low | ["Within-study bias","Imprecision","Incoherence"] |
| DTBT:Yoga | 0 | Some concerns | Low risk | No concerns | Major concerns | No concerns | Major concerns | Very low | ["Within-study bias","Imprecision","Incoherence"] |
| Mul_C:PMT | 0 | No concerns | Low risk | No concerns | Major concerns | No concerns | Major concerns | Low | ["Imprecision","Incoherence"] |
| PMT:Yoga | 0 | No concerns | Low risk | No concerns | Major concerns | No concerns | Major concerns | Low | ["Imprecision","Incoherence"] |

1. Huhn M, Nikolakopoulou A, Schneider-Thoma J, et al. Comparative efficacy and tolerability of 32 oral antipsychotics for the acute treatment of adults with multi-episode schizophrenia: a systematic review and network meta-analysis. *Lancet* 2019; **394**(10202): 939-51.

2. Higgins JPT, Jackson D, Barrett JK, Lu G, Ades AE, White IR. Consistency and inconsistency in network meta-analysis: concepts and models for multi-arm studies. *Res Synth Methods* 2012; **3**(2).

3. Dias S, Welton NJ, Caldwell DM, Ades AE. Checking consistency in mixed treatment comparison meta-analysis. *Stat Med* 2010; **29**(7-8): 932-44.

4. Puhan MA, Schünemann HJ, Murad MH, et al. A GRADE Working Group approach for rating the quality of treatment effect estimates from network meta-analysis. *BMJ* 2014; **349**.

5. Veroniki AA, Mavridis D, Higgins JPT, Salanti G. Characteristics of a loop of evidence that affect detection and estimation of inconsistency: a simulation study. *BMC Med Res Methodol* 2014; **14**: 106.
